# What is known about the protective factors that promote LGBTI+ youth wellbeing? A scoping review protocol

**DOI:** 10.12688/hrbopenres.13018.2

**Published:** 2020-06-10

**Authors:** Nerilee Ceatha, Marta Bustillo, Louise Tully, Oscar James, Des Crowley

**Affiliations:** 1 University College Dublin, Belfield, Dublin, D4, Ireland; 2 Royal College of Surgeons in Ireland, St Stephen's Green, Dublin, D2, Ireland; 3 Irish College of General Practitioners, Dublin, Co Dublin, D2, Ireland; 4 Health Services Executive, Dublin North Central, Co Dublin, D9, Ireland

**Keywords:** Youth, LGBTI+, sexual and gender minority, wellbeing, protective factors, scoping review

## Abstract

**Background:** There is much concern at the substantial vulnerabilities experienced by lesbian, gay, bisexual, transgender and intersex (LGBTI+) youth as a consequence of discrimination, stigmatisation and marginalisation. Recent research highlights the importance of understanding factors that can promote wellbeing for this population. This paper presents a protocol for a scoping review which aims to systematically map and synthesise the extent and nature of the peer-reviewed, published and unpublished academic literature on the protective factors that promote wellbeing for sexual and gender minority young people.

**Methods:** In accordance with the methodological framework for scoping reviews, the following six stages will be undertaken: (1) identifying the research question, (2) identifying relevant studies, (3) study selection, (4) charting the data, (5) collating, summarising and reporting results and (6) consultation. The PRISMA Extension for Scoping Reviews (PRISMA-ScR): Checklist and Explanation will be used throughout the review process. Key inclusion criteria will use the Population, Concept, Context approach, with two reviewers independently conducting the screening and extraction stages across five databases. Identified protective factors will be collated, summarised and categorised iteratively by one reviewer in consultation with the review team. Stakeholder consultation is a key strength of the scoping review process and will be complemented by the public patient involvement of LGBTI+ young people with expertise by experience.

**Conclusions:** The scoping review has the potential to inform policy, practice and future research through enhanced understandings of the complex interplay of factors that promote wellbeing for sexual minority, gender minority and intersex youth. This first stage of the research process will inform the development of a larger research project. The findings will be disseminated through a peer reviewed publication, a conference presentation and by sharing the findings with key stakeholders, including LGBTI+ young people.

## Introduction

It is accepted that there is “no health without mental health” ^1^, with the World Health Organization (WHO) describing mental health as “a state of wellbeing” ^2^. While the concept of wellbeing is contested, it is used extensively throughout the literature with less clarity about how this is defined ^3, 4^. This scoping review is informed by the WHO’s holistic conceptualisation of wellbeing ^2^. This encompasses a dynamic process that includes: life satisfaction, purpose and meaning; experiencing equilibrium, vitality and self-efficacy; alongside a sense of contribution ^2^. Furthermore, the WHO’s *Ottawa Charter of Health Promotion* situates individuals within their wider cultural and social environment, emphasising the importance of addressing the ecological context in order to strengthen the person ^5^. As such, social justice is a necessary pre-requisite for health ^5^. This conceptualisation facilitates a move beyond implicit understandings of a dichotomy of mental wellness and mental illness ^3, 4^. However, these binary understandings may be part of a wider public discourse where mental health has become synonymous with mental ill-health, as distinct from positive mental health or the relationship between mental health and social wellbeing ^3– 6^. Such understandings may be informed by a clinical and pathologised framing of mental health as referring solely to difficulties and concerns requiring diagnosis and treatment with resultant stigmatisation ^2– 4, 7– 9^. Research regarding attitudinal changes in the wider Irish population identified greater reluctance to openly share information about mental health concerns in personal and professional relationships ^7– 9^. Furthermore, over half the Irish population would not wish others to know if they had a mental health difficulty ^6^. In response, improved mental health and wellbeing have been identified as central to mental health promotion in Ireland with the launch of *Connecting for Life: Ireland’s national strategy to reduce suicide 2015–2020* ^10^. The Strategy outlines protective factors, such as help-seeking and social connectedness; however, the under-development of research into protective factors for mental health, particularly for population-specific target groups, is acknowledged ^10^.

While *Connecting for Life* adopts a whole-population approach, it identifies specific priority groups, notably those vulnerable to suicide including lesbian, gay, bisexual, transgender, intersex, questioning, queer, asexual, and non-binary (LGBTI+) populations ^10^. The acronym LGBTI+ comprises three dimensions: sexual orientation, gender identity and sex development. Sexual orientation refers to identification, behaviour and attraction, with suggestions of a higher life-time prevalence of same-sex behaviour and attraction than identification, and a higher reported prevalence of bisexual people, particularly bisexual women ^11^. Gender identity refers to someone’s internal sense of their gender as male, female or non-binary, and may not accord with the sex ascribed at birth ^11^. As such, those who identify as transgender and non-binary also have a sexual orientation and may be heterosexual, lesbian/gay, bisexual or asexual ^11^. Intersex refers to the spectrum of variations of sex development that occur within humanity ^12^. Likewise, those who identify as intersex also have a gender identity and sexual orientation. 

The wellbeing of LGBTI+ populations has generated considerable research interest over many decades, often identifying vulnerability to psychological distress, self-harm and suicide, both nationally and internationally ^10, 13^. This is often contextualised through Minority Stress Theory ^14^, highlighting the consequence of stigmatisation, marginalisation and discrimination on the wellbeing of LGBTI+ populations ^13– 15^. Particular concern has been expressed in relation to LGBTI+ youth with noted mental health disparities ^16– 19^. These concerns also extend to the Irish context ^10, 20– 22^.

There is a tendency to focus on resultant deficits, with sparse literature on strengths-based approaches which promote and protect LGBTI+ wellbeing ^23, 24^. This may assume that, because of the marginalisation of sexual minority, gender minority and intersex youth, these young people are automatically ‘at risk’ ^25, 26^. Researchers have cautioned against this continued representation which may inadvertently portray young LGBTI+ lives as a trajectory, assuming self-harm and suicidality ^27, 28^. It has been emphasised that the research taken to indicate heightened mental health risk fails to emphasise that the vast majority of those who identify as LGBTI+ report positive experiences of mental health and social wellbeing ^13, 25– 29^. Such generalised and universalising approaches may not recognise the range of mental health promoting factors available to LGBTI+ populations, including young people ^29, 30^. Furthermore, researchers have emphasised that the dominant research focus on mental health risk for LGBTI+ youth does not identify interventions or suggest solutions ^23, 24^. As a result, this representation may unintentionally stigmatise sexual minority, gender minority and intersex young people, with a subsequent decrease in help-seeking ^21, 31^. The national and international literature suggesting substantial vulnerabilities highlights the importance of understanding factors that are protective for this population ^10, 13, 21^.

The current research imbalance and emphasis on mental health disparities has been acknowledged ^10, 13, 31^. Health and social policies are integral to promoting the social acceptance of LGBTI+ people with stark differences noted between countries and over time ^32^. In particular, marked contrasts are evident amongst countries formerly colonised by Britain in relation to decriminalisation of homosexuality ^32^. This can be seen in the Irish context, where despite its history of colonisation, there has been significant progress since 1993 following decriminalisation ^33^. The rapid recognition of LGBTI+ rights was markedly enhanced in 2014 when the Irish Government become a signatory to the *Declaration of Intent* on the International Day Against Homophobia and Transphobia ^34^. This commitment informed legislative and policy measures including: a referendum and subsequent changes to the constitution to provide for marriage equality; legislation on gender recognition for adults; and the introduction of policy, including the publication of the *LGBTI+ National Youth Strategy* as part of the programme for government ^35– 37^. The strategy was the first of its kind in a global context, with three overarching goals with the third of these prioritising the development of the research and data environment ^37^. This acknowledges the dearth of research internationally and the urgent need for Irish-specific studies to ensure policy and practice is evidence-informed in relation to promoting LGBTI+ youth wellbeing ^37^.

It is within this context that this scoping review is being undertaken.

## Methods

### Study rationale

The scoping review is part of a broader PhD mixed-methods project. This first stage of the research process will locate the study within the literature to inform the future development of the qualitative and quantitative aspects of the project ^38, 39^. As far as we can determine there is no existing peer reviewed or published synthesis of the research on protective factors for LGBTI+ youth wellbeing. This is surprising, given the extensive research focus, over several decades, on LGBTI+ mental health disparities, with specific attention given to youth mental health risk ^10, 13, 16– 22^. The limited research focus on protective factors that promote LGBTI+ wellbeing is noteworthy given that a decade has passed since Haas and colleagues drew attention to the issue ^13^. This group of researchers specifically recommended that studies should be conducted on potentially protective factors, including those that mitigate mental health risk and promote resilience for sexual and gender minority populations ^13^.

### Scoping review objectives

A scoping review was chosen in order to ascertain the breadth of work conducted in this field ^40– 43^. In line with current recommendations, the overarching objective is to collect and synthesise the quantitative, qualitative and mixed-methods academic literature on the protective factors for sexual minority, gender minority and intersex youth wellbeing. The review aims to map the concepts, themes and types of available evidence within the existing national and international literature ^40^. The purpose of this scoping review is to make available the progress of this field of study, the characteristics of those studies undertaken to date, the various domains assessed, and the specific outcome measures used ^40^. Furthermore, this review will identify the extent and nature of the academic literature, including research deficits and knowledge gaps ^41^. The scoping review has the potential to inform policy, practice and future research, particularly through mapping a course forward to guide the planning and the commissioning of future research ^43^. A key strength of the scoping review framework is the priority placed on stakeholder consultation as part of the methodological framework ^40, 41, 43^. This suggests that both the process and results are strengthened through the contribution of those with research, policy and practice knowledge or expertise of the topic ^40, 41, 43^. A potential benefit of scoping reviews is that they can address topics considered important in the policy context ^42, 43^. The project is aligned to the Irish *LGBTI+ National Youth Strategy* and the specific objective to “develop research into the factors that support positive mental health for LGBTI+ young people” (p.31) ^37^. Stakeholders involved in the development and implementation of the Strategy will be invited to contribute to the consultation process. This will be complemented by consultation within broader LGBTI+ networks internationally. Furthermore, Daudt, van Mossel and Scott suggest that suitable stakeholders should be invited to be part of the research team ^43^. The study team includes members from within LGBTI+ communities with research, policy and practice backgrounds. The consultation will be enhanced by engaging LGBTI+ youth with expertise by experience via focus groups ^44– 46^.

The following research question will be considered: What is the extent (that is the size and breadth), range (variation and topic), and nature (characteristics, design, focus) of the evidence on the topic: ‘protective factors – LGBTI+ – youth – wellbeing’?

## Protocol design

Scoping reviews are defined as “a systematic approach to map evidence on a topic and identify main concepts, theories, sources and knowledge gaps” (p 467) ^47^. They assist in charting outcomes across settings and contexts, identifying where research is well-established and where there are gaps in the literature ^42^. The Preferred Reporting Items for Systematic Reviews and Meta-Analyses (PRISMA) Extension for Scoping Reviews (PRISMA-ScR): Checklist and Explanation will be used throughout the review process ^47^. The methodological framework will be followed as outlined by Arksey and O'Malley and Levac, Colquhoun and O’Brien ^40, 41^. This consists of six stages: (1) identifying the research question, (2) identifying relevant studies, (3) study selection, (4) charting the data, (5) collating, summarising and reporting results and (6) consultation ^40, 41^. While the sixth stage is considered optional within the framework, the authors concur with and Levac, Colquhoun and O’Brien that this process should be prioritised ^41^. This process offers the potential to enhance understandings of the complex interplay of protective factors for for sexual minority, gender minority and intersex youth ^44– 46^.

These stages of the scoping review will be discussed in further detail below.

### Stage 1: identifying the research question

Through an initial review of the literature, alongside consultation with key stakeholders within LGBTI+ research networks, the overarching research question is: what is the extent, range, and nature of the peer-reviewed academic qualitative, quantitative and mixed-methods evidence regarding LGBTI+ youth wellbeing? This question has been revised or refined through informal the consultation with key stakeholders, consistent with iterative and reflexive approach underpinning scoping reviews ^40, 41, 43^.

### Stage 2: identifying relevant studies

In order to identify relevant studies, the search strategy will be underpinned by key inclusion criteria based on the PRISMA checklist for scoping reviews using the Population, Concept, Context (PCC) screening criteria ^47^:

- P – Population: sexual minority, gender minority, intersex and non-binary youth
- C – Concept: protective factors that promote wellbeing
- C – Context: any country (or region) with broadly comparable social acceptance measures

With the limited research attention given to the topic, the scoping review will focus on published and unpublished, academic, peer reviewed research literature including quantitative, qualitative, and mixed-methods publications. Additionally, review articles will be included, such as: evidence syntheses, systematic reviews, and meta-analyses using quantitative, qualitative, and mixed-methods approaches, scoping reviews, narrative reviews, rapid reviews, and realist reviews. The authors are aware of the wealth of grey literature. However, it is well-documented that assessment of study quality and risk of bias are not considered to be part of the scoping review process, which is to describe the breadth and nature of the evidence ^42, 48^. To ensure that some grey literature is included, academic dissertations will be included along with peer reviewed journal articles. Three key repositories will be searched including the Networked Digital Library of Theses and Dissertations, Electronic Theses Online Service and DART Europe. Other types of publication will be excluded from the search (i.e., book chapters, conference abstracts, reports, policy briefs etc.) or publications in a language other than English.

Careful consideration has been given to search timeframes, particularly in light of the rapid changes that have occurred across the cultural and social landscape within the past ten years. However, a core function of a scoping review is to determine the coverage of a body of literature on a given topic ^40, 41, 43, 48^. Furthermore, Meyer’s publication of Minority Stress Theory in 2003 continues to have resonance ^14, 30^. On this basis, no time limits will be placed on the initial search and the literature from journal inception will be scoped. It is anticipated that assessing the timeline of publications on this topic, will enable these changes to be mapped ^40– 43^.

The scoping review will consider all research with sexual minority, gender minority and intersex youth ^49^. This study recognises that the LGBTI+ acronym has particular resonance in the Irish context, with its use evolving iteratively through consultative policy-making processes ^37^. However, there are wide variations in sexuality, gender identity and diverse sex development, and how this is expressed in relation to identities and orientations, particularly for youth ^49^. The use of the LGBTI+ acronym reflects the authors’ understanding of this diversity and ways of expressing these variations across a wide spectrum. While the search strategy has been informed by the work of Lee, Ylioja and Lackey, care has been taken to ensure that the search terms do not simply replicate their work ^49^. As such, the search strategy has been specifically tailored for this scoping review with deliberately wide search parameters using a broad spectrum of self-descriptors and related terms (see *Extended data*) ^50^.

Studies with LGBTI+ populations aged between 10 and 24 years will be included, consistent with the National Youth Strategy definition of youth ^51^. A criterion for inclusion is that the study specifically targets young people. While it is recognised that some highly relevant studies may have a mix of eligible/ineligible participants, this review will include studies where the mean age falls within the specified age range, that is, between 10 and 24 years.

A holistic view of wellbeing underpins this review in accordance with the WHO ^2, 5^. This acknowledges that while the dominant research focus has been on mental health risk, many of these studies will also include information about protective. This construct is further informed by the systematic review conducted by Wilson and Cariola and their recommendation of the need for strengths-based research into “resilience and protective factors for sexual and gender minority youth…across microsystems and macrosystems” (pp206–207) ^30^. They emphasise the importance of exploring the environmental, community and interpersonal characteristics of LGBTI+ youth “leading happy well-adjusted lives” (p206) ^30^. While the available frames of reference for LGBTI+ youth via the twin tropes of: “victims in need of tolerance and inclusion” and “just like everyone else” are problematised ^28^, this approach may inform more nuanced understandings of such protective factors for times of compromised wellbeing ^30^. Furthermore, the authors concur that Minority Stress Theory may provide a framework for exploring sexual minority, gender minority and intersex youth understandings of mental health promotion strategies and interventions ^30^. This is consistent with *Connecting for Life* which identified different protective factors for adolescent and adult populations, for example, help-seeking was positively associated with youth wellbeing ^10^.

In light of the continued criminalisation of homosexuality in some parts of the world, with an impact on research and obtaining data, the context will be limited to countries and regions where there are broadly comparable measures of social acceptance of LGBTI+ populations as measured by the Global Acceptance Index ^32^. While this may limit generalisablity, the rapid pace of change, as can be seen in the example of Ireland, may generate interest and be relevant to countries and regions where there is less acceptance ^32^.

***Search strategy.*** The search strategy was developed in consultation with the subject liaison librarian. A preliminary search was undertaken to establish whether existing scoping reviews had been conducted on the topic or in relation to protective factors for LGBTI+ youth wellbeing. A search for any reviews already conducted or aligning with the topic was undertaken across the Cochrane Database of Systematic Reviews, Campbell Collection, Epistemonikos and JBI Evidence Synthesis. When this yielded no results, the Database of Abstracts of Reviews of Effects from the National Institute for Health Research and PROSPERO, an international database of systematic reviews regarding health and social wellbeing, were subsequently searched. When no systematic, scoping or any other type of review was identified, an initial keyword search was piloted across three electronic databases: PubMed, PsycINFO and ASSIA. This informed further review of the search strategy with informal consultation undertaken with key stakeholders and LGBTI+ young people to ensure that deliberately broad keywords and search terms were included. The LGBTI+ acronym refers to those self-identifying as lesbian, gay, bisexual, transgender, intersex, questioning, queer, asexual and non-binary (LGBTI+). However, this umbrella terms is not limited to these forms of self-identification. This approach recognises the varied range of terms used to refer to sexual minority, gender minority and intersex populations with 67 LGBTI+ self-identifiers, and related terms used in the search strategy ^49^. To identify relevant studies, the following terms for sexual and gender minorities will be searched including, but not limited to (Title/Abstract LGBTI OR LGBTQ OR lesbian OR gay OR bisexual OR asexual OR pansexual OR transgender OR intersex OR non-binary OR queer OR “sexual minority” OR “gender minority” OR homosexuality OR bisexuality), combined using AND with terms used for those aged 10–24 years including (Title/Abstract youth OR young person OR “young people” OR “young adult” OR adolescent OR adolescence OR teen OR teenage OR teenager OR juvenile OR minors OR “emerging adult”), combined using AND with terms for protect including (Keyword resilient OR “protective factor”), combined using AND with terms for wellbeing including (key wellness OR wellbeing OR “mental health” OR “quality of life” OR “happiness with life” OR “life satisfaction”

The authors further revised and refined the search strategy through a MeSH database, main subjects, thesaurus terms, keywords and index headings and to identify databases for inclusion. An electronic search strategy will be used to identify databases of the published, peer-reviewed literature. Based on the pilot search, six electronic databases have been selected, containing academic, peer-reviewed journals for systematic searching from inception with relevance to medicine, nursing, psychology, allied health, social sciences and education including: PubMed, CINAHL, PsycINFO, ASSIA, ERIC International (Proquest) and Academic Search Complete. The search strategy for PubMed database can be found in the online supplementary material (see *Extended data*) ^50^.

### Stage 3: study selection

The search results will be stored in an electronic referencing system and duplicates removed. Studies will be included for abstract screening if they meet the criteria for Population, Concept, Context as outlined ^47^. Two reviewers will initially screen a small portion of the available evidence independently, to ensure the inclusion and exclusion criteria is clear, firstly screening by title and abstract, with discussion until there is complete agreement regarding inclusion. A third reviewer will be consulted until there is consensus regarding the inclusion and exclusion criteria. Abstracts will subsequently be assessed by two reviewers, independently, with discussion afterward until there is agreement. If a consensus cannot be reached further discussion will take place with a third reviewer until there is an agreed decision to include or exclude the study for analysis. Study selection will be based on *a priori* inclusion and exclusion criteria as outlined in Table 1 below ^47^. A copy of the screening template is available as *Extended data* ^50^.

**Table 1.** Inclusion and exclusion criteria of study selection ^47^.

Inclusion	Exclusion
• Study includes participants who self-identify as lesbian, gay, bisexual, transgender, intersex, queer, questioning, asexual, nonbinary or related terms	• Heterosexual, cisgender participants only • No demographic measure of sexual orientation, gender identity, non-binary or intersex status
• Study conducted in a country (or region) with a broadly similar Global Acceptance Index rank	• Study conducted in a country (or region) with a widely disparate Global Acceptance Index rank ^28^
• Study with participants aged 10–24 years • Study where young people are specifically targeted • Study whereby the mean age falls within the specified age range	• Study whereby participants are children ≤10 years or adults ≥24 years • Study whereby the mean age falls outside the specified age range
• Study referring to any measures of resilience • Study referring to ecological, psychosocial or cognitive measures that protect wellbeing OR • Study referring to minority stress	• No reference in study to resilience • No reference to any protective factors including: interpersonal, community-based or policy measures OR • No reference to factors that mitigate minority stress
• Published in English	• Published in language other than English
• Peer-reviewed	• Non-peer-reviewed
• Academic journal article or dissertation	• Book, book chapter, conference abstract, paper or keynote, report etc.

Queries for discussion will be recorded using a written template (see *Extended data*) ^50^. A table with excluded studies and reasons for exclusion will be generated based on this *a priori* criteria. The study team will be consulted throughout this process of selecting sources of evidence. The final step includes citation searching of the studies where the reference lists of the included articles will be reviewed using the citation function of Google Scholar to search for additional studies, supplemented by reference searching ^40, 41, 43, 47^.

### Stage 4: charting the data

The data extraction process in scoping reviews is referred to as ‘charting’ the data ^40, 41, 43, 47^. A data extraction tool will be developed and piloted by the research team to provide a written overview of the key information to be captured in a summary table ^47^. This will record the following information: author(s); year of publication; country where study was conducted; aims/purpose of the study; methodology/methods; study population and sample size; outcomes and key findings in relation to the scoping review questions. The project lead will complete a full-text review and extract the data according to the summary table. All variables will be identified including assumptions and simplifications. The summary table will be tested by the study team to check a random sample of the completed data extracted. Consultation will take place with the study team throughout this process. This will establish processes for obtaining and confirming data. Where there is disagreement, the study team will be consulted until there is a decision to include or exclude a study. It is anticipated that completion of the summary table will be an iterative and reflexive process ^40, 41, 43, 47^.

### Stage 5: collating, summarising and reporting the results

The PRISMA-ScR checklist for reporting scoping reviews will be used ^47^. A rationale for collating the results will describe the methods and the use of this information in any data synthesis will be outlined ^47^. The methods of handling and summarising the charted data will be described ^47^. The selection of sources of evidence will be outlined using a flow diagram ^47^. The characteristics of sources of evidence will be described and cited, with the results synthesised and summarised ^47^. It is anticipated that there will be variation in study design and method. A general interpretation will be provided along with potential implications and limitations of the scoping review ^47^.

### Stage 6: consultation

***Stakeholder consultation.*** A scoping review provides for a consultation stage as part of the methodological framework ^40^. Levac, Colquhoun and O’Brien recommend that this stage is a requirement for a scoping review as it enhances methodological rigor ^41^. The consultation will be undertaken with stakeholders recruited through a purposive sampling approach ^52^. This widely used technique is considered particularly useful in identifying and selecting research and policy stakeholders who have particular knowledge about the research question ^52^. The review will seek to consult with those involved with the Irish *LGBTI+ National Youth Strategy*, either in its development of implementation phases ^37^. Those included in the consultation, will potentially include: representatives of national statutory and voluntary bodies within the education, health and social care and youthwork sectors; practitioners in health, social care, education and youthwork; researchers; and policy makers ^37^. This recognises that consultation enhances the scoping review process and that the results are more useful when stakeholders contribute to the work ^41, 43^. In order to embed consultation throughout the scoping review process, suitable stakeholders have been invited to be part of the research team. Daudt, van Mossel and Scott suggest this adds depth to all stages of the review ^43^. The purpose of the stakeholder consultation comprises three phases: (1) search phase: reviewing and revising search terms and inclusion/exclusion criteria; (2) analysis phase: an iterative refinement of included sources of evidence and relevant data (3) findings: critical appraisal to ensure correct data interpretation and suggestions for knowledge translation ^41, 43^. This will provide an opportunity to provide insights beyond those in the literature in order to analyse, interpret and integrate the study findings ^41, 43^.

This will be enhanced by public patient involvement (PPI) through focus groups with LGBTI+ young people.

***Patient and public involvement.*** Involvement of young people in research processes is increasingly emphasised by researchers, policy makers and practitioners ^45– 46^. The provision within the framework for a consultation stage aligns with the objectives of the study and recognises LGBTI+ youth expertise, the social and cultural capital within youth networks and youth agency regarding their mental health and wellbeing ^45– 46^. PPI approaches recognise that the inclusion, engagement and participation of young people in research is both child-centred and rights-based ^46^. Focus groups will use interactive participatory approaches to share the findings from the scoping review to explore LGBTI+ young people’s understandings, meanings and interpretations ^45^. In Ireland, it is acknowledged that some young people are seldom-heard, including sexual and gender minority youth ^44^. Baker and Beagan call for collaboration that promotes ‘learning *with’* LGBTI+ communities ^53^. This recognises that young people can and should be more involved in all stages of the research process, not just in providing data to researchers ^44– 46^. The preliminary findings from the stage 5 framework will inform the consultation ^41^. This will inform further synthesis of the review.

## Dissemination and ethics

Ethical approval has been granted from the UCD Humanities Research Ethics Committee to undertake consultations on the scoping review findings with stakeholders and to conduct focus groups with young people (Ref#: HS-19-80-Ceatha-Campbell ). It is hoped that the involvement of sexual minority, gender minority and intersex youth will provide insights into research dissemination with these populations of young people, their families and with professionals in health, social care and education ^46^. The research team anticipate that the results will be disseminated through a peer reviewed publication, a conference presentation and presentations to the key stakeholders, including LGBTI+ youth.

## Discussion

To our knowledge, this will be the first scoping review exploring the protective factors that promote the wellbeing of sexual minority, gender minority and intersex youth, including young people who self-identify as LGBTI+. Drawing on Arksey and O'Malley’s methodological framework, the search strategy will map the established quantitative, qualitative and mixed-methods evidence of the protective factors currently considered, identify research gaps and provide recommendations ^40^. The PRISMA Extension for Scoping Reviews (PRISMA-ScR) checklist will be used throughout the review process in order to ensure transparency and enhancing the potential for replication ^47^. Stakeholder consultation is a key strength of the scoping review process and will engage with a range of people with expertise in research, policy and practice ^41, 43^. This will be complemented by PPI with sexual minority, gender minority and intersex youth through interactive, participatory focus groups ^45^. This offers a unique opportunity to include LGBTI+ young people with expertise by experience in the research process ^46^. A recognised limitation of scoping reviews is that they do not assess the quality of studies, like the more traditional systematic review. However, a scoping review methodology has the capacity to collect a great quantity of different types of literature. This is of particular relevance when there is little known or published about a topic area. This scoping review has the potential to ascertain the work conducted in the field, map the available literature and identify key concepts ^43^. This could inform the design of future full systematic reviews on this subject area by demonstrating the breadth and nature of the literature to date ^43, 48^.

## Data availability

### Underlying data

No underlying data are associated with this article.

### Extended data

Open Science Framework: Scoping review protocol on protective factors that promote LGBTI+ youth wellbeing. DOI 10.17605/OSF.IO/6PMDA ^50^.

This project contains the following extended data:

- Version 2 PubMed search string scoping review OSF (DOCX). (PubMed search string to be used in the scoping review.)
- Version 2, Screening criteria using Population, Concept, Context for OSF (DOCX). (Screening criteria to be used in the scoping review.)
- Version 2, Table 1. Inclusion and exclusion criteria of study selection for OSF (DOCX). (Screening criteria to be used in the scoping review.)
- Version 2 Screening template of inclusion and exclusion criteria OSF (DOCX). (Eligibility criteria screening template to be used in the scoping review.)
- Flow chart inclusion & exclusion criteria OSF (PPT). (Flow chart of decisions for inclusion and exclusion for the scoping review.)

Extended data are available under the terms of the Creative Commons Zero "No rights reserved" data waiver (CC0 1.0 Public domain dedication).

## References

[1] Prince M Patel V Saxena S: No health without mental health. *Lancet.* 2007;370(9590):859–77. 10.1016/S0140-6736(07)61238-0 17804063

[2] World Health Organization: The world health report: 2001: Mental health: New understanding, new hope.Geneva: WHO.2001;1 Reference Source

[3] Dooris M Farrier A Froggett L: Wellbeing: the challenge of ‘operationalising’ an holistic concept within a reductionist public health programme. *Perspect Public Health.* 2018;138(2):93–99. 10.1177/1757913917711204 28574301

[4] Dodge R Daly AP Huyton J: The challenge of defining wellbeing. *International Journal of Wellbeing.* 2012;292(3). 10.5502/ijw.v2i3.4

[5] World Health Organization: Ottawa charter for health promotion: an International Conference on Health Promotion, the move towards a new public health. World Health Organization: Canada.1986;17–21. 10.1093/heapro/1.4.405

[6] Barry M van Lente E Molcho M: SLÁN 2007: Survey of lifestyle, attitudes and nutrition in Ireland: Mental health and social well-being report. Dublin: Department of Health and Children, The Stationery Office.2009 Reference Source

[7] Millward Brown Landsdown: Irish attitudes towards mental health problems. Dublin: See Change.2012.

[8] Millward Brown Landsdown: Public attitudes towards mental illness: A benchmark study for See Change. Dublin: See Change.2010.

[9] Mac Gabhann L Lakeman R McGowen P: Hear my Voice: The experience of discrimination of people with mental health problems in Ireland. Dublin: Dublin City University.2010 Reference Source

[10] National Office for Suicide Prevention: Connecting for life: Ireland’s national strategy to reduce suicide 2015-2020. Health Services Executive, Dublin.2014 Reference Source

[11] Gates G, Williams Distinguished Scholar: How many people are lesbian, gay, bisexual, and transgender?UCLA, CA: The Williams Institute.2011 Reference Source

[12] Roen K: Intersex or diverse sex development: Critical review of psychosocial health care research and indications for practice. *J Sex Res.* 2019;56(4–5):511–28. 10.1080/00224499.2019.1578331 30907687

[13] Haas AP Eliason M Mays VM: Suicide and suicide risk in lesbian, gay, bisexual, and transgender populations: review and recommendations. *J Homosex.* 2010;58(1):10–51. 10.1080/00918369.2011.534038 21213174 PMC3662085

[14] Meyer IH: Prejudice, social stress, and mental health in lesbian, gay, and bisexual populations: conceptual issues and research evidence. *Psychol Bull.* 2003;129(5):674–697. 10.1037/0033-2909.129.5.674 12956539 PMC2072932

[15] HSE National Social Inclusion Governance Group: LGBT Health: Towards meeting the health care needs of lesbian, gay, bisexual and transgender people. Dublin: HSE.2009 Reference Source

[16] Liu RT Sheehan AE Walsh RFL: Prevalence and correlates of non-suicidal self-injury among lesbian, gay, bisexual, and transgender individuals: A systematic review and meta-analysis. *Clin Psychol Rev.* 2019; 101783. 10.1016/j.cpr.2019.101783 31734440 PMC6896220

[17] Lucassen MF Stasiak K Samra R: Sexual minority youth and depressive symptoms or depressive disorder: A systematic review and meta-analysis of population-based studies. *Aust N Z J Psychiatry.* 2017;51(8):774–787. 10.1177/0004867417713664 28565925

[18] Marshal MP Dietz LJ Friedman MS: Suicidality and depression disparities between sexual minority and heterosexual youth: a meta-analytic review. *J Adolesc Health.* 2011;49(2):115–23. 10.1016/j.jadohealth.2011.02.005 21783042 PMC3649127

[19] Zhao Y Montoro R Igartua K: Suicidal ideation and attempt among adolescents reporting “unsure” sexual identity or heterosexual identity plus same-sex attraction or behavior: forgotten groups? *J Am Acad Child Adolesc Psychiatry.* 2010;49(2):104–13. 10.1097/00004583-201002000-00004 20215932

[20] McMahon EM O’Regan G Corcoran P: Young Lives in Ireland: a school-based study of mental health and suicide prevention. Cork, National Suicide Research Foundation.2017 10.13140/RG.2.2.21820.03208

[21] Higgins A Doyle L Downes C: The LGBTIreland Report: national study of the mental health and wellbeing of lesbian, gay, bisexual, transgender and intersex people in Ireland. Dublin, GLEN/BeLonGTo.2016 Reference Source

[22] GLEN/BeLonGTo: Key Findings: LGBT Lives.2009 Reference Source

[23] Gahagan J Colpitts E: Understanding and Measuring LGBTQ Pathways to Health: A Scoping Review of Strengths-Based Health Promotion Approaches in LGBTQ Health Research. *J Homosex.* 2017;64(1):95–121. 10.1080/00918369.2016.1172893 27043161

[24] Herrick AL Stall R Goldhammer H: Resilience as a research framework and as a cornerstone of prevention research for gay and bisexual men: theory and evidence. *AIDS Behav.* 2014;18(1):1–9. 10.1007/s10461-012-0384-x 23321946

[25] Bryan A Mayock P: Speaking back to dominant constructions of LGBT Lives: Complexifying ‘at riskness’ for self-harm and suicidality among lesbian, gay bisexual and transgender youth. *Irish Journal of Anthropology.* 2012:15(2):8 Reference Source

[26] Bryan A Mayock P: Supporting LGBT Lives? Complicating the suicide consensus in LGBT mental health research. *Sexualities.* 2017;20(1–2M):65–85. 10.1177/1363460716648099

[27] Savin-Williams RC: The new gay teenager. (Vol. 3). Cambridge, Massachussetts, Harvard University Press.2005 10.4159/9780674043138

[28] Talburt S Rofes E Rasmussen ML: Introduction: Transforming Discourses of Queer Youth and Educational Practices Surrounding Gender, Sexuality, and Youth. In: M. L. Rasmussen, E. Rofes & S. Talburt (Eds.), *Youth and sexualities: Pleasure, subversion, and insubordination in and out of schools.*New York: Palgrave Macmillan.2004;1–13. 10.1057/9781403981912_1

[29] Fredriksen-Goldsen KI Simoni JM Kim HJ: The Health Equity Promotion Model: Reconceptualization of Lesbian, Gay, Bisexual, and Transgender (LGBT) Health Disparities. *Am J Orthopsychiatry.* 2014:84(6):653–663. 10.1037/ort0000030 25545433 PMC4350932

[30] Wilson C Cariola LA: LGBTQI+ Youth and Mental Health: A Systematic Review of Qualitative Research. *Adolesc Res Rev.* 2019;1–25. 10.1007/s40894-019-00118-w

[31] Roberts LM Marx RA: The persistence of policies of protection in LGBTQ research & advocacy. *J LGBT Youth.* 2018;15(4):280–299. 10.1080/19361653.2018.1479325

[32] Flores AR: Social Acceptance of LGBT People in 174 Countries 1981 to 2017. Williams Institute: UCLA.2019 Reference Source

[33] Kuhl M: Ireland wins LGBT+ activism award at World Pride in New York. In Gay Community News; NXF Dublin, Ireland.2019 Reference Source

[34] International Day Against Homophobia and Transphobia (IDAHOT). Declaration of Intent. 2014 Reference Source

[35] Attorney General of Ireland: Marriage Act 2015.Irish Statute Book; Oireachtas: Dublin, Ireland.2015 Reference Source

[36] Attorney General of Ireland: Gender Recognition Act 2015.Irish Statute Book; Oireachtas: Dublin, Ireland.2015 Reference Source

[37] Department of Children and Youth Affairs (DCYA): LGBTI+ National Youth Strategy.DCYA, The Stationery Office: Dublin.2018 Reference Source

[38] Creswell JW Creswell JD: Research design: Qualitative, quantitative, and mixed methods approaches.SAGE publications: Los Angeles.2019;31(1):75–77. 10.1002/nha3.20258

[39] Mertens D Ginsberg P: Deep in Ethical Waters: Transformative Perspectives for Qualitative Social Work Research. *Qual Soc Work.* 2008;7(4):484–503. 10.1177/1473325008097142

[40] Arksey H O'Malley L: Scoping studies: towards a methodological framework. *Int J Soc Res Methodol.* 2005;8(1):19–32. 10.1080/1364557032000119616

[41] Levac D Colquhoun H O’Brien KK: Scoping studies: advancing the methodology. *Implement Sci.* 2010;5(1):69. 10.1186/1748-5908-5-69 20854677 PMC2954944

[42] Chang S: Scoping Reviews and Systematic Reviews: Is It an Either/Or Question? *Ann Intern Med.* 2018:169(7):502–503. 10.7326/M18-2205 30178036

[43] Daudt HM van Mossel C Scott SJ: Enhancing the scoping study methodology: a large, inter-professional team’s experience with Arksey and O’Malley’s framework. *BMC Med Res Methodol.* 2013;13(1):48. 10.1186/1471-2288-13-48 23522333 PMC3614526

[44] Tricco AC Lillie E Zarin W: PRISMA Extension for Scoping Reviews (PRISMA-ScR): Checklist and Explanation. *Ann Intern Med.* 2018;169(7):467–473. 10.7326/M18-0850 30178033

[45] Kelleher C Seymour M Halpenny AM: Promoting the Participation of Seldom Heard Young People: A Review of the Literature on Best Practice Principles.Department of Children and Youth Affairs, Dublin.2014 10.21427/D7GF69

[46] McEvoy O: A practical guide to including seldom heard children and young people in decision-making.Department of Children and Youth Affairs, Dublin.2015 Reference Source

[47] Lundy L: ‘Voice’ is not enough: conceptualising Article 12 of the United Nations Convention on the Rights of the Child. *Br Educ Res J.* 2007;33(6):927–942. 10.1080/01411920701657033

[48] Munn Z Peters MDJ Stern C: Systematic review or scoping review? Guidance for authors when choosing between a systematic or scoping review approach. *BMC Med Res Methodol.* 2018;18(1):143. 10.1186/s12874-018-0611-x 30453902 PMC6245623

[49] Lee JG Ylioja T Lackey M: Identifying lesbian, gay, bisexual, and transgender search terminology: A systematic review of health systematic reviews. *PLoS One.* 2016;11(5):e0156210. 10.1371/journal.pone.0156210 27219460 PMC4878791

[50] Ceatha N: Scoping review protocol on protective factors that promote LGBTI+ youth wellbeing. 2020 10.17605/OSF.IO/6PMDA PMC7295080 32566894

[51] Department of Children and Youth Affairs: National Youth Strategy 2015–2020.Dublin: Government Publications.2015 Reference Source

[52] Rubin HJ Rubin IS: Qualitative Interviewing: The art of hearing data.Los Angeles, CA Sage Publications.2005 Reference Source

[53] Baker K Beagan B: Making assumptions, making space: an anthropological critique of cultural competency and its relevance to queer patients. *Med Anthropol Q.* 2014;28(4):578–598. 10.1111/maq.12129 25196115

