# Supplementary material for: What is known about the protective factors that promote LGBTI+ youth wellbeing? A scoping review protocol
Source: HRB Open Res. 2020 Jun 10;3:11. Originally published 2020 Mar 30. [Version 2] doi: 10.12688/hrbopenres.13018.2 (PMC7295080; doi:10.12688/hrbopenres.13018.2)
Supplement: Peer review history

# Peer review: Reviewer response for version 2

**DOI:** 10.21956/hrbopenres.14184.r27528

**Published:** 2020-06-12

**Authors:** Yael Perry

**Affiliations:** 1 Telethon Kids Institute, Perth, Australia

I have reviewed the authors’ responses to my comments and am satisfied that they have addressed my concerns. Happy to approve the protocol.

Is the study design appropriate for the research question?

Yes

Is the rationale for, and objectives of, the study clearly described?

Yes

Are sufficient details of the methods provided to allow replication by others?

Partly

Are the datasets clearly presented in a useable and accessible format?

Not applicable

Reviewer Expertise:

Youth mental health, LGBTIQ mental health, prevention of mental illness.

I confirm that I have read this submission and believe that I have an appropriate level of expertise to confirm that it is of an acceptable scientific standard.

# Peer review: Reviewer response for version 1

**DOI:** 10.21956/hrbopenres.14111.r27304

**Published:** 2020-05-27

**Authors:** Michelle Johns

**Affiliations:** 1 Division of Adolescent and School Health, Centers for Disease Control and Prevention, Atlanta, GA, USA

This review addresses an important gap in the literature, and I applaud the research team for taking it on.

 Considerations:

 Wellbeing is a broad construct that has been operationalized in many ways. It would be helpful for the authors to define how they are operationalizing wellbeing within the introduction to help establish the boundaries of this review for the reader. 

 Additionally, the inclusion of "mental health" as a key word is likely to pull many articles related to mental health disorders/sequalae such as depression, anxiety, PTSD, etc. It would be useful to address head on how the research team will handle such articles. Do they fit the scope? If so, under what circumstances? 

 The authors may be interested to know that a prior systematic review focused on the issue of transgender youth and protective factors, and included considerations of mental health: Johns, M.M., *et al.*, (2018) ^1^.

Is the study design appropriate for the research question?

Yes

Is the rationale for, and objectives of, the study clearly described?

Yes

Are sufficient details of the methods provided to allow replication by others?

Yes

Are the datasets clearly presented in a useable and accessible format?

Not applicable

Reviewer Expertise:

Sexual and gender minority health, sexual health, mental health, stigma, resilience.

I confirm that I have read this submission and believe that I have an appropriate level of expertise to confirm that it is of an acceptable scientific standard.

## References

[1] : Protective Factors Among Transgender and Gender Variant Youth: A Systematic Review by Socioecological Level. *J Prim Prev*.39(3) : 10.1007/s10935-018-0508-9 263-301 10.1007/s10935-018-0508-9 29700674PMC5976555

# Response

**Published:** 2020-06-05

**Authors:** Nerilee Ceatha

**Affiliations:** University College Dublin, Ireland

We would like to thank the reviewer for their approval of the scoping review. We also welcome the suggestions of how the protocol can be improved, especially by clarifying constructs and how these will be operationalised. We will respond to these points below:

Wellbeing

We agree with the suggestion that it would be helpful to define wellbeing and how this will be operationalised in the introduction. Revising the protocol has provided an opportunity to make this more explicit in order to help establish the boundaries of the review. We accept that wellbeing is contested ^1,2^. Our use of the term  draws from the holistic framing by the WHO ^3^. This is further complemented by situating individuals within an ecological model where health is premised on social justice ^4^. To operationalise this construct we have drawn on the work of Wilson and Cariola and made this specific within the inclusion criteria ^5^.

Mental health

We view mental health as an integral component of wellbeing. This approach is premised on our understandings that mental health is not binary; that anyone may experience times when their mental health may be compromised and that this does not preclude times of wellbeing ^1,2^. This holistic conceptualisation attempts to move away from clinical and pathologized framing of mental health which can be stigmatising ^1^. Careful consideration has been given regarding articles including articles related to mental health diagnoses and sequalae. Again, building on Wilson and Cariola’s review, this has been operationalised using Minority Stress Theory ^5^. As such, the scoping review will include articles which refer to mental health and then specifically review to determine whether there is any reference to factors that mitigate minority stress including, but not limited to: interpersonal, community-based or policy measures, alongside measures of resilience. To operationalise this, we have developed a flow chart of inclusion and exclusion criteria and further revised the screening tool (see *Extended data*) ^6^.

Prior systematic review

We are aware of the systematic review by Johns et al on transgender youth and protective factors ^7^.  We have been using this study as a reference when piloting the search strategy to ensure that the search captures key articles, reviews such as this.

We again thank the peer reviewer for their review. We very much appreciate the feedback and believe that the revised protocol has been strengthened by addressing these suggestions which will be beneficial throughout the scoping review process.

1. Dooris, M, Farrier, A, & Froggett, L. Wellbeing: the challenge of ‘operationalising’ an holistic concept within a reductionist public health programme. *Perspectives in Public Health*, 2018, *138*(2), 93-99.
2. Dodge R, Daly AP, Huyton J, Sanders LD. The challenge of defining wellbeing. International Journal of Wellbeing. 2012, *29*;2(3).
3. World Health Organization (WHO). *Mental health atlas 2005*. A Report by the Department of Mental Health and Substance Dependence. World Health Organization: Geneva, 2005.
4. World Health Organization. *Ottawa charter for health promotion: an International Conference on Health Promotion, the move towards a new public health.* 17-21 November, World Health Organization: Canada, 1986.
5. Wilson, C, & Cariola, LA. LGBTQI+ Youth and Mental Health: A Systematic Review of Qualitative Research. *Adolescent Research Review*, 2019, 187-211.
6. Ceatha N: Scoping review protocol on protective factors that promote LGBTI+ youth wellbeing. 2020. http://www.doi.org/10.17605/OSF.IO/6PMDA
7. Johns, MM, Beltran, O, Armstrong, HL, Jayne, PE, & Barrios, LC. Protective factors among transgender and gender variant youth: A systematic review by socioecological level. *Journal of Primary Prevention*. 2018, *39*(3), 263-301.

# Peer review: Reviewer response for version 1

**DOI:** 10.21956/hrbopenres.14111.r27305

**Published:** 2020-04-20

**Authors:** Yael Perry

**Affiliations:** 1 Telethon Kids Institute, Perth, Australia

This scoping review protocol outlines a plan to conduct a very important and timely piece of research that has clear implications for the field, as well as practice and policy. It is well conceptualised and situated in a clear context. The focus on protective factors is most welcome in a sea of literature focused on risk factors. Nevertheless, there are some clarifications around constructs and criteria that I believe would further improve the protocol and the review itself, which I have outlined below:

- The authors have not included grey literature in the scope of the literature they intend to review. Given the breadth of a scoping review, and the potential to uncover relevant and meaningful data in the grey literature, it is advisable that this addition is considered
- The authors refer to ‘stakeholders’ throughout the protocol but do not define who these people are. It would be helpful to describe who the stakeholders are and what ‘stake’ they hold in relation to the research
- The definition of LGBTI+ provided is very limited. The dimensions of sexual orientation and gender identity do not encompass the ‘I’ for intersex (physical diversity), and the examples of both gender (male/female/non-binary) and sexual diversity (hetero/lesbian/gay/bisexual/asexual) do not encompass the myriad of identities and orientations people regularly express. The authors should clarify that a very wide spectrum of sexual orientations and gender identities exist (and are subjectively defined) and that the examples provided are not exhaustive. Consider reviewing (and using) the following systematic review on terminology in your search terms: Lee, J. G., Ylioja, T., & Lackey, M. (2016) ^1^.
- With regards to the research question, the authors oscillate between using the terms ‘protective factors’ and ‘youth wellbeing’. This needs to be clarified as wellbeing is a very vague construct and potentially encompasses far more than simply protective factors. The current operationalization of the wellbeing concept includes mental health – is this the opposite of mental illness or something else? I suggest that protective factors is used as the primary Concept (C) in place of wellbeing as this is far more specific and arguably will lend itself to a more useful review.
- Delete repeated references to the social science liaison librarian – this may have been important for the development of your conceptualization of the review, but isn’t usually included in the protocol.
- No time limits are placed on the literature search, however, the social context has changed dramatically for LGBTI+ young people over recent years. Is it appropriate to review and include literature from the 80s or 90s?
- The exclusion criteria for age (less than 50% of participants are in the specified age range) may be practically difficult to use – most studies won’t state participants’ ages by %. May want to consider using the mean age falling within the specified age range (as well as a criterion that specifies the study targets young people specifically, so as not to inadvertently include a study targeting adults that happens to recruit a young population).
- The authors should consider using a standardized risk of bias tool (e.g. Mixed Methods Assessment Tool which can be used for both quantitative and qualitative studies).

An additional (minor) point – for the sake of clarity and ease of reading - some of the protocol is repetitive in nature. It would benefit from an additional review by the authors to minimize this repetition.

Is the study design appropriate for the research question?

Yes

Is the rationale for, and objectives of, the study clearly described?

Yes

Are sufficient details of the methods provided to allow replication by others?

Partly

Are the datasets clearly presented in a useable and accessible format?

Not applicable

Reviewer Expertise:

Youth mental health, LGBTIQ mental health, prevention of mental illness.

I confirm that I have read this submission and believe that I have an appropriate level of expertise to confirm that it is of an acceptable scientific standard, however I have significant reservations, as outlined above.

## References

[1] : Identifying Lesbian, Gay, Bisexual, and Transgender Search Terminology: A Systematic Review of Health Systematic Reviews. *PLoS One*.2016;11(5) : 10.1371/journal.pone.0156210 e0156210 10.1371/journal.pone.0156210 27219460PMC4878791

# Response

**Published:** 2020-06-05

**Authors:** Nerilee Ceatha

**Affiliations:** University College Dublin, Ireland

We would like to thank the reviewer for their detailed review and helpful feedback. We particularly appreciate the constructive comments of how the protocol can be improved, especially by clarifying constructs and criteria. We will respond to each of the points below:

Grey literature

We have given careful consideration to the wealth of grey literature and its inclusion. While we concur that there is a wealth of grey literature, there is broad variation in the quality of that literature. However, critical appraisal is not part of a scoping review methodology and this review would not include quality assessment and risk of bias ^1,2,3^. We acknowledge that there may potentially be a lot of important grey literature in dissertation form. We have now included dissertations, which may be unpublished, and will search three key repositories.

Stakeholders

Thank you for highlighting the need to describe and define the stakeholders who have been and will be invited to consult throughout the protocol development and scoping review process. Informed by the work of Levac and colleagues, we have provided further details of the purpose for the consultation and sought to specify the stakeholders invited to consult ^1^. Drawing on the recommendations of Daudt and colleagues, we have also clearly articulated that the study team includes suitable stakeholders ^3^.

Use of the LGBTI+ acronym

In relation to the use of the LGBTI+ acronym, we welcome these suggestions. We are aware of the wide variations and how this is expressed in relation to identities and orientations regarding sex, gender and sexual orientation. This review is being conducted within the Irish policy context, with LGBTI+ being the preferred acronym ^4^. However, we wish to ensure that this review has international resonance. Thank you for drawing attention to the importance of ensuring that definitions of diversity of sexual orientations and gender identities should be expanded to encompass other expressions, including intersex identifications ^5^. We have made this more explicit throughout the protocol. Further, we have highlighted that the use of broad search parameters reflects our understanding of this diversity and ways of expressing these variations across a wide spectrum. While the work of Lee, Ylioja and Yackey was cited, this has now been made more explicit ^6^.

Refining the Research Question re: wellbeing

Revising the protocol has provided an opportunity to ensure consistency and outline how constructs will be operationalised with regards to the research question. We now refer to ‘protective factors’ throughout the protocol. We have given consideration to the use of ‘wellbeing’ as the Primary Concept (C) and agree that this can be a vague term, however, it is used extensively throughout the literature. As such, much would be lost if the search did not include this term. However, we concur that the inclusion of the term ‘wellbeing’ needs to be specific in order to provide a more useful review. We have amended the Primary Concept to ‘protective factors that promote wellbeing’. We have clarified that our use of the term  ‘wellbeing’ draws from a holistic concept as outlined by the WHO ^7^, complemented by the Ottawa Charter for health promotion ^8^. We have drawn on the work of Wilson and Cariola and specifically outlined how these constructs will be operationalised within the inclusion and exclusion criteria. We welcome the question on the inclusion of mental health, which we view as an integral component of wellbeing. This approach is premised on our understandings that mental health is not binary; that anyone may experience times when their mental health may be compromised and that this does not preclude times of wellbeing ^9,10^. This holistic conceptualisation attempts to move away from clinical and pathologized framing of mental health which can be stigmatising ^9^. Again, building on Wilson and Cariola’s review, this has been operationalised in the context of Minority Stress Theory ^11^. As such the scoping review will include articles which refer to mental health and then specifically screen to determine whether there is any reference to factors that mitigate minority stress including, but not limited to: interpersonal, community-based or policy measures, alongside measures of resilience. To operationalise this, we have developed a flow chart of inclusion and exclusion criteria and further revised the screening tool (see *Extended data*) ^12^.

Reference to the subject librarian

The repeated references have been removed.

Timeframe on the search

We have given careful consideration as to whether to impose a timeframe on the search. One of the core functions of a scoping review is to determine the coverage of a body of literature on a given topic ^2^. While it is acknowledged that there have been rapid changes in the last decade, with the example of Ireland one of the most dramatic in this regard,  assessing the timeline of publications on this topic will facilitate the mapping of these changes ^3^. Further, Wilson and Cariola recommend that Minority Stress Theory, published in 2003, may be useful in addressing research gaps ^11^.

Inclusion and exclusion criteria regarding age

Thank you for the suggestion that we include a criterion that specifies the study targets young people and that the mean age of study participants should fall within the specified age range. This is very helpful. The inclusion and exclusion criteria have been amended to reflect these suggestions.  

Risk of bias

A ‘standardized risk of bias tool’ would be appropriate for use with a systematic review, rather than a scoping review ^2^. Thus, assessment using a ‘standardized risk of bias tool’ is beyond the scope of this review.  Further, these types of review have the potential to determine whether a systematic review is needed and the potential design of this. Daudt and colleagues suggest that scoping reviews may be a “necessary pre-requisite” for determining whether a full systematic review is required ^3^.

Repetition

The protocol has been edited to address the repetition in the writing.

Thank you for the review and the encouraging comments that the study is timely and important with implications for research, policy and practice. We very much appreciate the feedback and believe that the revised protocol has been strengthened by addressing these suggestions. These refinements and revisions will be beneficial throughout the scoping review process.

References

1. Levac D, Colquhoun H, O’Brien KK. Scoping studies: advancing the methodology. *Implementation Science.* 2010, *5*(1):69.
2. Munn, Z., Peters, M.D.J., Stern, C. *et al.* Systematic review or scoping review? Guidance for authors when choosing between a systematic or scoping review approach. *BMC Medical Research Methodology. *2018, *18*,143. 
3. Daudt HM, van Mossel C, & Scott SJ. Enhancing the scoping study methodology: a large, inter-professional team’s experience with Arksey and O’Malley’s framework. *BMC Medical Research Methodology. *2013, *13*(1):48.
4. Department of Children and Youth Affairs (DCYA). LGBTI+ National Youth Strategy; DCYA, The Stationery Office: Dublin, 2018.
5. Roen K. Intersex or diverse sex development: Critical review of psychosocial health care research and indications for practice. *The Journal of Sex Research.* 2019 Jun 13;56(4-5):511-28.
6. Lee JG, Ylioja T, Lackey M: Identifying lesbian, gay, bisexual, and transgender search terminology: A systematic review of health systematic reviews. *PLoS One.* 2016;11(5):e0156210. 27219460 10.1371/journal.pone.0156210 4878791
7. World Health Organization (WHO). *Mental health atlas 2005*. A Report by the Department of Mental Health and Substance Dependence. World Health Organization: Geneva, 2005.
8. World Health Organization. *Ottawa charter for health promotion: an International Conference on Health Promotion, the move towards a new public health.* 17-21 November, World Health Organization: Canada, 1986.
9. Dooris, M, Farrier, A, & Froggett, L. Wellbeing: the challenge of ‘operationalising’ an holistic concept within a reductionist public health programme. *Perspectives in Public Health*, 2018, *138*(2), 93-99.
10. Dodge R, Daly AP, Huyton J, Sanders LD. The challenge of defining wellbeing. International Journal of Wellbeing. 2012, *29*;2(3).
11. Wilson, C, & Cariola, LA. LGBTQI+ Youth and Mental Health: A Systematic Review of Qualitative Research. *Adolescent Research Review*, 2019, 187-211.
12. Ceatha N: Scoping review protocol on protective factors that promote LGBTI+ youth wellbeing. 2020. http://www.doi.org/10.17605/OSF.IO/6PMDA
